# An Analysis of the Sequence Variability of Meningococcal fHbp, NadA and NHBA over a 50-Year Period in the Netherlands

**DOI:** 10.1371/journal.pone.0065043

**Published:** 2013-05-24

**Authors:** Stefania Bambini, Jurgen Piet, Alessandro Muzzi, Wendy Keijzers, Sara Comandi, Lisa De Tora, Mariagrazia Pizza, Rino Rappuoli, Diederik van de Beek, Arie van der Ende, Maurizio Comanducci

**Affiliations:** 1 Novartis Vaccines and Diagnostics, Siena, Italy; 2 Academic Medical Center, Department of Medical Microbiology, Amsterdam, The Netherlands; 3 The Netherlands Reference Laboratory for Bacterial Meningitis, Amsterdam, The Netherlands; 4 Academic Medical Center, Department of Neurology, Amsterdam, The Netherlands; Health Protection Agency, United Kingdom

## Abstract

Studies of meningococcal evolution and genetic population structure, including the long-term stability of non-random associations between variants of surface proteins, are essential for vaccine development. We analyzed the sequence variability of factor H-binding protein (fHbp), Neisserial Heparin-Binding Antigen (NHBA) and *Neisseria* adhesin A (NadA), three major antigens in the multicomponent meningococcal serogroup B vaccine 4CMenB. A panel of invasive isolates collected in the Netherlands over a period of 50 years was used. To our knowledge, this strain collection covers the longest time period of any collection available worldwide. Long-term persistence of several antigen sub/variants and of non-overlapping antigen sub/variant combinations was observed. Our data suggest that certain antigen sub/variants including those used in 4CMenB are conserved over time and promoted by selection.

## Introduction

*Neisseria meningitidis* is a major cause of invasive bacterial meningitis and septicemia worldwide. Meningococcal populations are highly diverse, and engage in extensive genetic exchange [1], [2]. Studies of genetic variation, by MultiLocus Enzyme Electrophoresis (MLEE) [3] and subsequently by MultiLocus Sequence Typing (MLST) [4], show that meningococci are structured into clonal complexes [5], [6]. Isolates belonging to the different clonal complexes exhibit different phenotypes and vary in their propensity to cause disease [7], [8], [9]. Meningococcal isolates belonging to the hypervirulent clonal complexes (cc)32, cc11, cc8, cc41/44, cc1, cc4 and cc5, have a high capacity to cause invasive disease. Also cc269 could be considered hypervirulent [10]. It is genetically related to cc32, as shown by whole genome analysis [11]. Strains belonging to the hypervirulent clonal complexes are over-represented in collections of pathogenic isolates. Clonal complexes are relatively genetically stable over time, despite high rates of recombination [4]. In contrast, strains associated with asymptomatic carriage exhibit more extensive genetic diversity [12].

The paradoxical persistence of clonal complex structure despite high levels of recombination can be explained by evolutionary models that invoke positive selection [13], [14], [15], which has important implications for the design of protein-based vaccines against meningococcal serogroup B (MenB). Outer membrane vesicle (OMV) vaccines that rely on the immunogenic properties of PorA [16], [17], [18], [19] have been used: though, those vaccines only provide protection for homologous strains carrying the same PorA [20]–[23]. To overcome antigenic variability, vaccines based on multiple outer membrane proteins have been proposed to provide protection against a broad range of meningococcal isolates. Novel antigens were identified by Reverse Vaccinology [20], [21], [22] and combined into a multicomponent vaccine against MenB, 4CMenB (Bexsero®),, which has been recently licensed in the Europe [23], [24], [25]. 4CMenB includes OMV from the New Zealand MeNZB® vaccine [26] and three major protein antigens: factor H-binding protein (fHbp), Neisserial Heparin-Binding Antigen (NHBA) and *Neisseria* adhesin A (NadA).

fHbp (genome-derived *Neisseria* antigen [GNA] 1870 or LP2086) [27], [28] binds human factor H [29], [30], enhancing the ability of the bacterium to resist complement-mediated killing. Its expression enables survival in *ex vivo* human blood and serum [31], [32], [33]. As different fHbp classification schemes have been proposed, a dedicated database is available (http://neisseria.org/nm/typing/fhbp), with a unified fHbp nomenclature for the assignment of new sub-variants. fHbp has been classified into three (main) variants 1, 2 and 3, described here as fHbp-1, fHbp-2 and fHbp-3 [28], which were further divided into sub/variants fHbp-1.x, fHbp-2.x and fHbp-3.x, where x denotes the specific peptide sub/variant. In a different nomenclature scheme, the sub/variants are grouped into subfamily A (corresponding to variants 2 and 3) and subfamily B (corresponding to variant 1) based on sequence diversity [27]. Bactericidal activity is variant specific; antibodies raised against one variant are not cross-protective against other variants, even if some cross-reactivity has been described between fHbp-2 and -3 [28]. Antibodies raised against sub/variant fHbp-1.1, included in 4CMenB vaccine, are highly cross-reactive with fHbp-1 and poorly cross-reactive with fHbp-2 and -3 [34]. Clinical trials are also ongoing for a different vaccine including fHbp-1.55 and fHbp-3.45 [35], [36].

NHBA (GNA2132) is a heparin-binding protein [37] which shows sufficient sequence diversity to prevent a subdivision into a small number of variant families [38], [39]. For this reason NHBA nomenclature is based on the assignment of a unique identifier x to each specific peptide sub/variant, which was indicated as NHBA-x. The 4CMenB vaccine contains NHBA peptide 2 (NHBA-2), which elicited antibodies that were cross-reactive with heterologous sub/variants and cross-protective in the infant rat model [40], [41].

NadA (GNA1994) plays a role in cell adhesion and invasion [42]. The *nadA* gene is present in almost all isolates belonging to the hypervirulent lineages cc8, cc11 and cc32, and in all strains so far isolated belonging to cc213 and cc1157 [46]–[48]. Conversely, it is absent in almost all strains belonging to cc41/44 and cc269. NadA protein is present in five variants. NadA-1, NadA-2 and NadA-3 elicit cross-reactive bactericidal antibodies [43]. NadA is usually absent in carrier isolates, although a small proportion express NadA-4, which does not induce cross-reactive bactericidal antibodies to NadA-1, NadA-2 and NadA-3 [44]. NadA-5 occurs mostly in cc213 strains [38], [45]. The NadA protein included in 4CMenB is NadA-3 sub/variant 8 (NadA-3.8), which induces antibodies cross-reactive with NadA-1, NadA-2 and NadA-3 [43].

Statistical association studies indicate that the repertoire of fHbp, NHBA and NadA is structured among hyperinvasive lineages. Isolates from the same clonal complexes have similar profiles for each antigen, even when derived from disparate geographical locations and time periods [38].

We investigated the prevalence and sequence variation of fHbp, NHBA and NadA in a panel of 165 pathogenic meningococcal isolates randomly selected from those collected in the Netherlands over a period of 50 years.

## Results

### Prevalence and Distribution of Sequence Types (STs) and MLST Clonal Complexes Over Time

Ninety-five different STs and 22 clonal complexes were found (**Table S1**).

**Figure 1:**
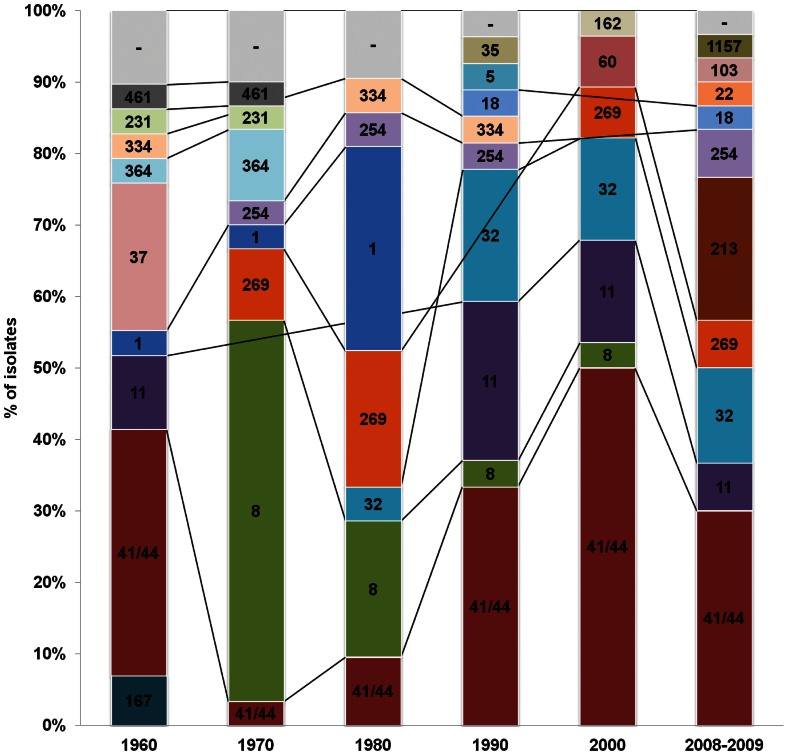
Distribution of the MLST clonal complexes from 1960 to 2008-2009 in the Netherlands. Clonal complexes (cc)1, cc11, cc32, cc231, cc254, cc269, cc334, cc41/44 and cc8 persisted for more than two decades. cc41/44 (27%), cc8 (13%), cc11(9%), cc32(8%) and cc269 (7%) were the most frequent. cc41/44, which was present in all time-periods considered, was predominant in 1960, 1990, 2000 and 2008-2009, cc8 was predominant in 1970 and cc1 in 1980. cc5, cc22, cc35, cc37, cc60, cc103, cc162, cc167, cc213, cc1157 were observed only once, cc18, cc364, cc461 were present twice. Over time, there were several ccs that disappeared, such as cc37 and cc167,which were present in 1960 only, and on the other new ccs emerged.

ST-8 (9%), ST-11 (8%), ST-41 (8%), ST-1 (5%) were the most commonly represented STs. ST-1, ST-41 and ST-11 were the only STs detected in more than two consecutive decades based on the sample studied. In contrast, 82 (86%) STs were observed only once, 7 (7%) twice and 3 (3%) in two or three non consecutive decades. Twenty-seven percent of the isolates belonged to cc41/44, 13% to cc8, 9% to cc11, 8% to cc32 and 7% to cc269. The remaining 36% of isolates included 17 different clonal complexes. Ten clonal complexes were observed only once, 3 were present twice, whereas 9 persisted for more than two decades (**Fig. 1**).

### Diversity and Distribution of fHbp

The *fHbp* gene was present in all isolates. Forty-nine different nucleotide sequences were identified. Forty-five out of the 47 different amino acid sequences were in frame. 98% of amino acid sequences belonged to the three variants: fHbp-1 (52%), fHbp-2 (39%) and fHbp-3 (7%). Concerning the remaining 2%, in one case the fHbp variant was a natural chimera between fHbp-1 and fHbp-3, which was recently described [30]; one sequence presented a frame-shift, causing a premature end of the encoded peptide; moreover, in one case the gene sequence was not obtained (**Table S1**).

**Figure 2:**
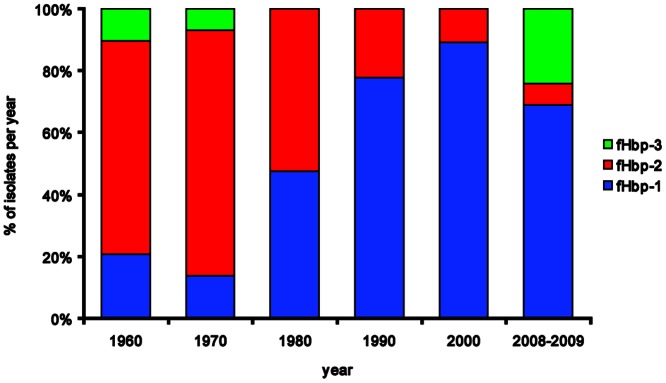
Distribution of the three fHbp variants Over Time. fHbp-2 predominated in 1960-1980 and was subsequently substituted by fHbp-1. The presence of fHbp-3 in 2008-2009 was mainly related with the emergence of the cc213.

**Figure 3:**
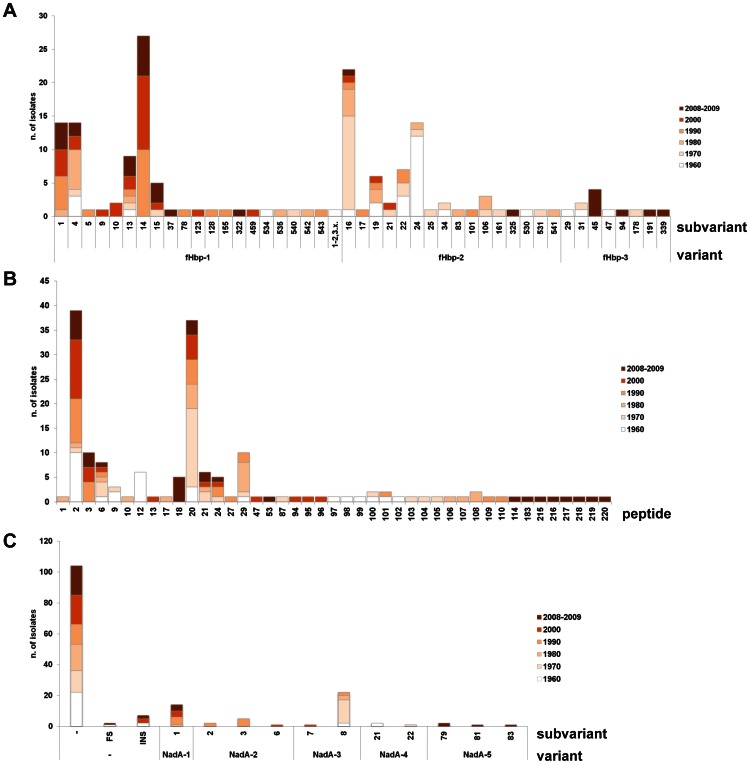
Distribution of antigens from 1960 to 2008-2009. **A) fHbp variants and sub/variants.** Out of the 45 sub/variants in frame identified from 1960 to 2008-2009, fHbp-1.14 (16%), 2.16 (13%), 1.1 (8%), 1.4 (8%), and 2.24 (8%) were the most frequent and persisting. fHbp-2.24 was predominant in 1960, 2.16 in 1970, 1.4 in 1980, 1.14 in 1990, 2000 and 2008-2009, together with 1.1. **B) NHBA peptides.** Out of the 43 peptides identified from 1960 to 2008-2009, NHBA- 2 (24%) and 20 (22%) were the most frequent and persisting. NHBA-2 was predominant in 1960, 1990, 2000 and 2008-2009, NHBA-20 in 1970 and 1980 (this latter case, together with NHBA-29). **C) NadA presence, variants and sub/variants.** *nadA* gene was absent or presented frame-shift mutations or insertion sequences in 115 out of 165 isolates. Out of the 11 sub/variants identified from 1960 to 2008-2009 in the 50 isolates with the gene, NadA-3.8 (40%) and NadA-1.1 (28%) were the most frequent and persisting. NadA-3.8 was present from 1960 to 1990, NadA-1.1 from 1980 to 2008-2009.

fHbp-2 predominated in 1960-1980, whereas fHbp-1 became more prevalent thereafter (**Fig. 2**). fHbp-1.14 (16%), 2.16 (13%), 2.24 (8%), 1.4 (8%), 1.1 (8%; included in 4CMenB), 1.13 (5%), 2.22 (4%), 2.19 (4%) and 3.45 (2%) were the most frequent sub-variants (**Fig. 3A**).

### Diversity and Distribution of NHBA

The *nhba* gene was found in all isolates considered. In one case the gene presented a frame-shift mutation, probably causing a lack of protein expression. Forty-eight different nucleotide sequences, corresponding to 43 different amino acid sequences, were identified (**Table S1**). NHBA-2 (24%), 20 (22%), 3 (6%), 29 (6%), 6 (5%), 12 (4%), 21 (4%), 18 (3%) and 24 (3%) were the most frequently occurring (**Fig. 3B**). NHBA-2 (included in 4CMenB) was predominant in 1960 (34%), 1990 (33%), 2000 (43%) and 2008-2009 (20%), whereas NHBA-20 was predominant in 1970 (53%) and NHBA-29 in 1980 (28%).

### Diversity and Distribution of NadA

Of 165 isolates, 60 (36%) had the *nadA* gene. Eight isolates had the *nadA* gene interrupted by the insertion sequence IS*1301.* Therefore, they most likely did not express the protein [43]. The gene of two isolates presented a frame-shift mutation. Therefore, 50/165 (30%) isolates were considered positive for NadA. The gene was absent in cc41/44 and cc269 with one exception in both clonal complexes, strain 600091 and 701844, respectively (**Table S1**). Variants NadA-1, NadA-2, NadA-3 and NadA-5, which are generally present in pathogenic strains, accounted for 28%, 16%, 42% and 8% of the positive isolates, respectively. NadA-4, the variant described in carrier strains, was found in 6% of the positive isolates. NadA-3 and NadA-4 were most frequent in 1960-1970, NadA-1 and NadA-2 were prevalent in 1990-2000, NadA-1 and NadA-5 in 2008-2009. Eleven sub/variants were identified (**Table S1**), the most frequent being NadA-3.8, the NadA sub-variant in 4CMenB, present in 20 out of the 50 isolates with the *nadA* gene (40%), which was predominant in 1960-’70-’80. NadA-1.1 (28%) has been prevalent after 1990 (**Fig. 3C**).

### Association of fHbp, NadA, and NHBA diversity with MLST Clonal Complexes

We observed clear associations between fHbp, NadA, and NHBA unique sequences with clonal complexes. These results, also obtained in other strain panels [38], were based on a standardized measure of association, the Cramer’s V coefficient (**Table 1**). The value of this coefficient of correlation is high, in particular for fHbp and NHBA (V_NHBA_ = 0.769, V_fHbp_ = 0.704 and V_NadA_ = 0.582).

**Table 1:** Associations between different loci.

	Measure of association with clonal complexes (Cramer’s V§)	Measure of the association between two different loci (Standardized Index of Association§, I_A_^S^)			Measure of non-overlapping structure of combinations (f* metrics§)		
Gene	Housekeeping genes	*fHbp*	*nhba*	*nadA*	*fHbp*	*nhba*	*nadA*
*fHbp*	0.705		0.371	0.667		0.284	0.601
*nhba*	0.769	0.371		0.512	0.284		0.462
*nadA*	0.582	0.667	0.512		0.601	0.462	

The Cramer’s V coefficient was used to measure association statistics with clonal complexes. The values indicated a strong association between antigen alleles and clonal complexes. The Standardized Index of Association *I_A_^S^* was used to test the stability of associations between different loci. The antigen pairs showed relatively high *I_A_^S^* values, indicative of strong, stable associations between the different loci over time. Moreover, the combinations showed a non-overlapping structure (measured by f* metrics) that suggested the immune selection maintaining antigenic combinations.

§ The three statistical parameters V, *I_A_^S^* and f* are based on the frequency of the alleles and vary between 0 (random distribution) and 1 (perfect association or non overlapping distribution in the case of f*).

Several sub/variants were associated with specific clonal complexes. For example, fHbp-1.1 was always present in cc32 isolates. Twenty-five out of 27 fHbp-1.14 were present in cc41/44 (92%). fHbp-2.16 was almost always represented in cc8 isolates (91%). fHbp-1.15 and fHbp-3.45 were found mostly in cc269 (80%) and cc213 (75%), respectively. However, eleven different fHbp sub/variants were found in cc41/44, the most frequent being fHbp-1.14 (55%). fHbp-2.16 was almost always represented in cc8 isolates. Seven and 9 different sub/variants were represented within cc11 and cc269, respectively.

Similar association with clonal complexes was observed for NHBA peptides. NHBA-3 was always present (100%) in cc32, NHBA-12 in cc37, NHBA-18 in cc213 and NHBA-21 in cc269. NHBA-2 was predominant in cc41/44 (37 out of 39 isolates, 95%). NHBA-29 was found mostly in cc1 (80%). NHBA-20 was the most frequent peptide in cc8 (57%), even if it was also present in cc11 (32%).

Also NadA variants were associated with clonal complexes. NadA-1 was solely present in cc32 strains. NadA-2 (7 out of 8 isolates, 88%) was mostly present in cc11 strains. NadA-3 (81%) was mostly present in cc8 strains. NadA-5 was solely present in cc213 strains.

### Evolution of cc41/44

**Figure 4:**
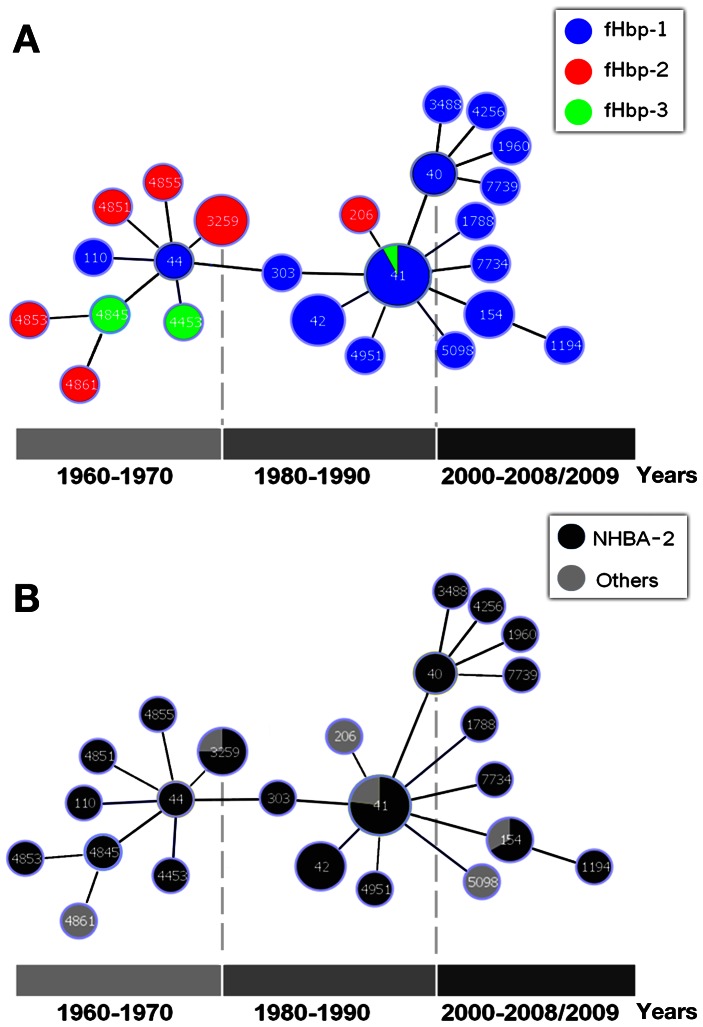
The evolution of cc41/44 over 50 years. The structure of cc41/44 was analysed with phyloviz based on the goeBURST algorithm [62]. Each number correspond to a different ST, the sizes of circles are proportional to the number of isolates. **A) fHbp variants.** In 1960-1970, when the ST-44 sub-complex was predominant, fHbp-2 and fHbp-3 were most represented. After 1980, when the ST-41 sub-complex was prevalent, fHbp-1 became the most frequent. Of note, independently from its prevalence, fHbp-1 was the only variant that was present in both sub-complexes at all time periods. **B) NHBA**. NHBA-2, the most frequent in cc41/44 (82%), was equally shared by the two sub-complexes from 1960 to 2008-2009.

The only clonal complex present at all observed time points in the sample was cc41/44. It showed a shift over time between its two central STs [6] (**Fig. 4**). The ST-44 sub-complex was predominant in 1960-1970, and ST-41 was prevalent after 1980.

Still, due to association between (any) protein diversity and MLST, a trend in the ratio of fHbp variants was observed to follow changes in the central genotypes (**Fig. 4A**). From 1960 to 1980, when the ST-44 sub-complex was predominant, fHbp-2 was most represented, and sub/variants fHbp-2.24 and 2.19 were predominant. After 1980, as sub-complex ST-41 became predominant, fHbp-1 became the most frequent variant and sub/variant fHbp-1.14 predominated after 1990 (**Table S1 and** **Fig. 3A**).

Similar associations with each of the two sub-complexes of cc41/44 were also observed for PorA and FetA variants. PorA P1.7-2, 4 and FetA F1-5 were associated with the ST-41 sub-complex isolates collected in 1990 and afterward, while this combination was not observed among the ST-44 sub-complex isolates. The latter was more heterogeneous with PorA P1.18, 25-7 prevalent.

In contrast, NHBA-2 was the most frequently occurring peptide in both cc41/44 sub-complexes across the 50-year period examined (**Fig. 4B**). As for all clonal complexes, the association with ST-41 and ST-44 sub-complexes, within cc41/44, was evaluated using Cramer’s V coefficient. NHBA-2 was predominant but evenly distributed between the two sub-complexes and consequently NHBA did not show a relevant enrichment of specific peptides in one of the sub-complexes (V_NHBA_ = 0.298). In contrast, the overall distribution of the fHbp sub/variants in cc41/44 was not uniform and showed a relevant association with sub-complexes (V_fHbp_ = 0.683).

### Long-term Persistence of Antigen Sub/variants

**Figure 5:**
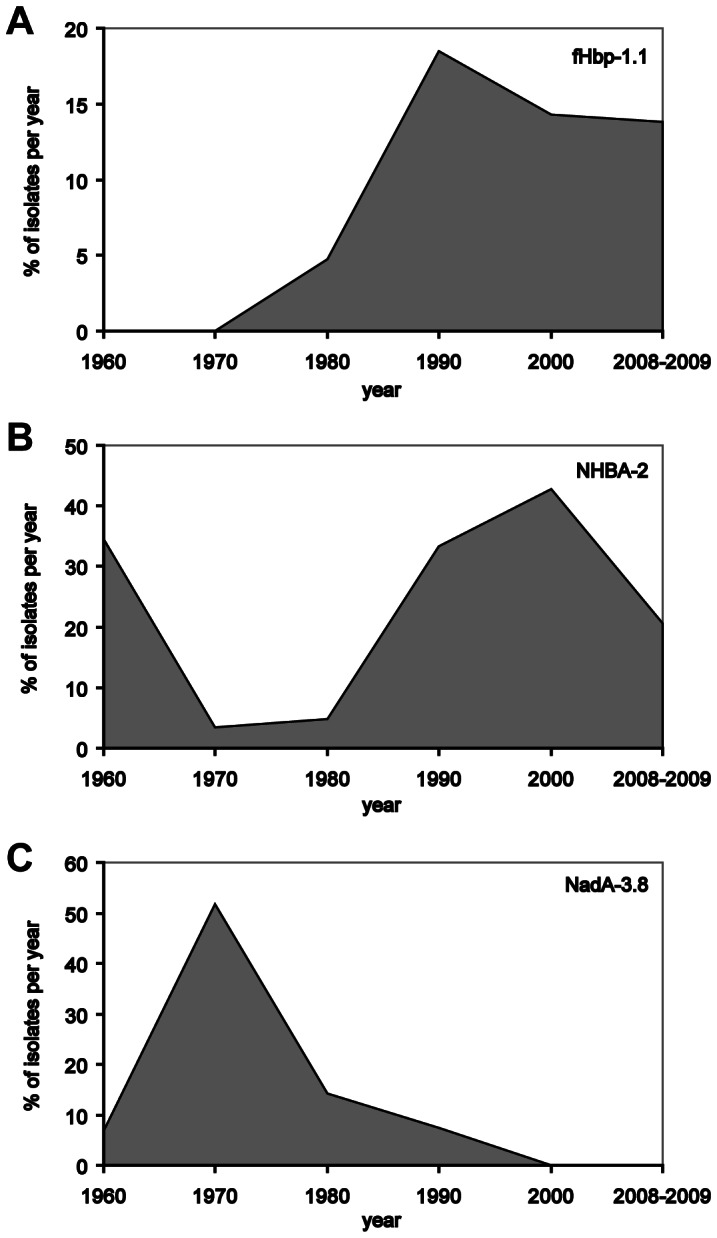
Persistence of the fHbp, NHBA and NadA sub/variants included in 4CMenB. fHbp-1.1 and NadA-3.8 persisted for thirty years, NHBA-2 for fifty years.

A number of antigen sub/variants appeared in the dataset only once, indicating a low frequency in the population. This might suggest that they were less fit and excluded by selection over time, while other sub/variants persisted over decades. Of the 45 fHbp sub/variants identified in the current sample, 30 were observed once, and 10 were seen over a period of at least 20 years. Among the 43 NHBA peptides observed, 30 occurred once, and 7 have been encountered over at least 20 years. Eleven NadA sub/variants were identified, of which 5 were observed once and 2 were present over at least 20 years. The proteins included in 4CMenB (fHbp-1.1 and NadA-3.8) were observed over thirty years, while NHBA-2 was observed over fifty years (**Fig. 5**).

In general, sub/variants that were found persisting over decades in this present study were found also in other strain panels from different time periods [38], [39], [45]. Moreover, several sub/variants have been associated over time also with different clonal complexes. For instance fHbp-1.13 was identified in cc254 strains collected during 1970, 1980, 1990 and 2008-2009, and in cc60 strains in 2000. fHbp-1.14 was identified in cc41/44 isolates in 1990, 2000 and 2008-2009, and in cc254 isolates collected in 2008-2009. fHbp-2.16 was found in cc8 isolates collected between 1970 and 2000, and in cc22 isolates from 2008-2009.NHBA-20 was identified in cc11 isolates in 1960, 1990, 2000 and 2008-2009, while in 1970 and 1980 it was prevalent in cc8.

### Long-term Persistence of Antigen Sub/variant Combinations

**Figure 6:**
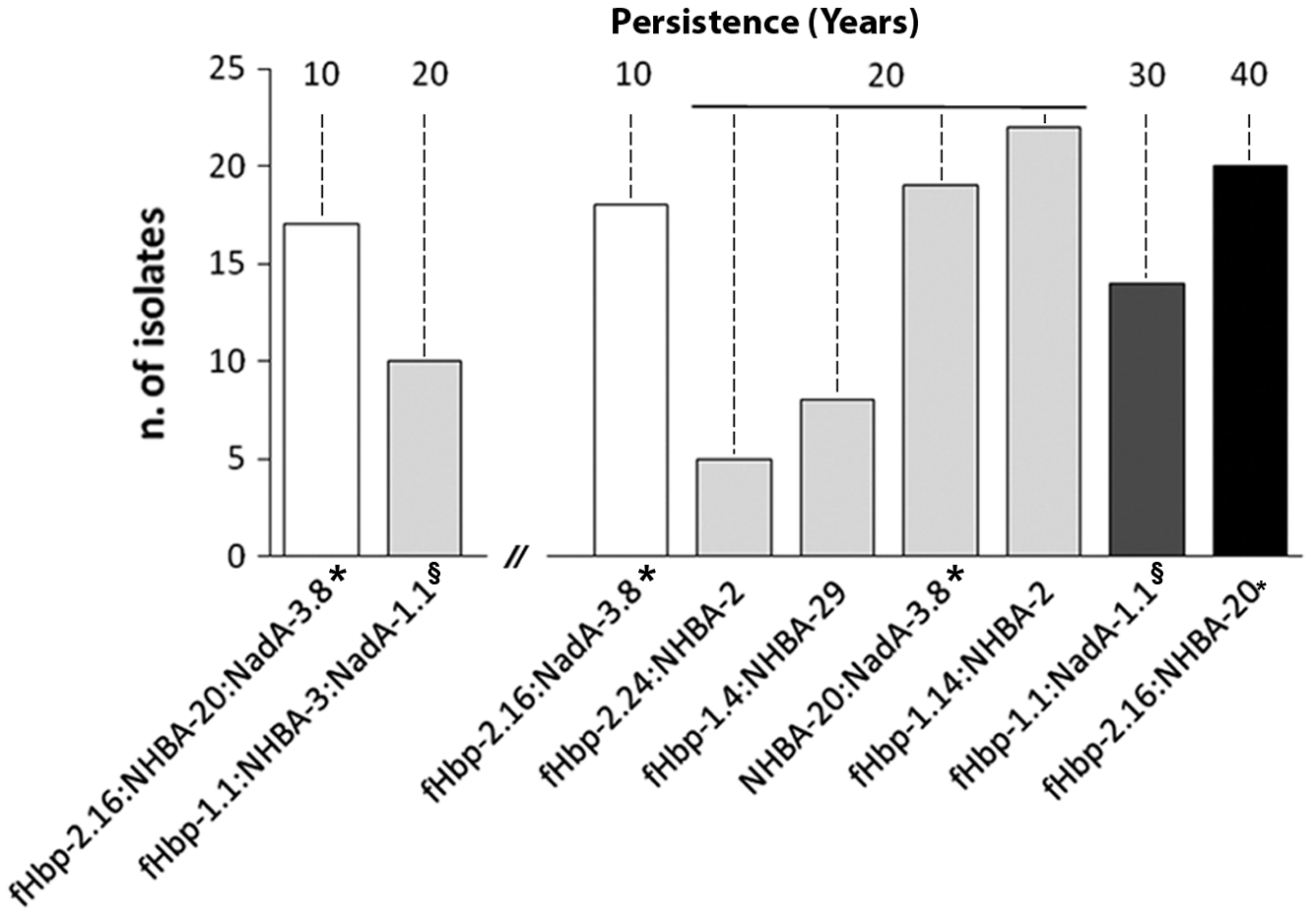
Longevity of the most frequent combinations of two and three antigen sub/variants. Out of the 9 most frequent combinations observed, several persisted for at least twenty years. fHbp-1.1:NadA-1.1 and fHbp-2.16:NHBA-20 were very stable, persisting for thirty and forty years, respectively. Combinations generated by fHbp-2.16, NHBA-20 and NadA-3.8 were indicated by the symbol (*). Combinations generated by fHbp-1.1, NHBA-3 and NadA-1.1 were indicated by the symbol (§).

Of 83 combinations of fHbp, NadA, and NHBA sub/variants**,** most were found only once, yet certain combinations were stable and persisted over time. Examples include: fHbp-1.1:NadA_-_1.1, which persisted for 30 years; and fHbp-2.16:NHBA-20, which persisted for 40 years (**Fig. 6**). The relative presence of several combinations changed over time. For example, fHbp-2.16:NHBA-20 was the most frequent in 1970, fHbp-1.4:NHBA-29 was more commonly found in 1980, and fHbp-1.14:NHBA-2 was predominant after 1990.

In addition to association of antigens sub/variants with clonal complexes, several antigen sub/variant combinations were maintained in the same clonal complex. The fHbp-1.1:NadA-1.1 combination, first observed in 1980, persisted in all isolates of cc32 for thirty years. The fHbp-1.14:NHBA-2 combination was found in cc41\44 isolates in 1990 and persisted for 20 years. The fHbp-1.4:NHBA-29 combination was found in cc1 isolates from 1960 to 1980. The fHbp-2.16:NHBA-20:NadA-3.8 combination was observed in cc8 isolates in 1970 and in 1980.

The Standardized Index of Association *I_A_^S^* [46] was used to test the stability of associations between pairs of loci, i.e. the presence of linkage disequilibrium (**Table 1**). The *I_A_^S^* of the *fHbp* with respect to *nhba* and *nadA* was 0.371 and 0.667 respectively, the index between *nhba* and *nadA* was 0.512. These three values showed a particular faculty of *nadA* to be associated with *fHbp* and *nhba*. For example, these rates were comparable with the same index calculated for the association between *porA* VR1 and VR2 (*I_A_^S^*  = 0.566), two very close loci belonging to the same gene. The linkage disequilibrium between the *fHbp*, *nhba* and *nadA* loci showed also a non-overlapping structure (measured by f*metrics, **Table 1**). The non-overlapping structure, in particular between *fHbp* and *nadA* (f*metrics  = 0.601) can be interpreted as the result of the pressure of selection exerted by the immune system in maintaining antigenic combinations in the neisserial population [13].

## Discussion and Conclusions

The sequence variability of fHbp, NHBA and NadA have been examined in several strain panels worldwide and investigations are still ongoing [38], [39], [45]. We evaluated prevalence and sequence variations of fHbp, NHBA and NadA in a panel of invasive meningococcal isolates collected in the Netherlands from 1960 to 2009, which, is to our knowledge, the widest time frame of any collection worldwide.

Among the meningococci isolated in a 50 year period many sub/variants of each antigen fHbp, NHBA and NadA were identified. Although most sub/variants were observed only once, or persistent over a period of a few years, a significant number, such as those used in 4CMenB, were observed to persist over time. Of note, the antigen sub/variants we found to be stable and conserved over at least twenty years have been identified as the most frequent in other panels of invasive isolates [38]-[42], [47], [48]. Short-lived sub/variants were likely less fit or occurred too infrequently to be observed, which may suggest that they were excluded by immune selection.

Associations between antigen sub/variants and clonal complexes measured by the Cramer’s V coefficient, a standardized measure of association, were also identified and were consistent with previous studies in other strain panels [38], [45].

The three protein sub/variants chosen for inclusion in 4CMenB (fHbp-1.1, NHBA-2 and NadA-3.8) were the most common worldwide and were expected to provide cross-reactivity [31], [33], [37], [41]. An interesting and unexpected finding of this study is their long-term persistence in the current strain panel, which was thirty years for fHbp-1.1 and NadA-3.8, and fifty years for NHBA-2.

Long term stability and persistence was observed also for several combinations of protein sub/variants. Antigen combinations showed a non-overlapping structure. As reported in literature, if immune selection was absent or neutral, then all antigen variants and sub/variants would occur for similar time spans. On the contrary, it has been suggested that selection may cause strong linkage disequilibrium (or non-random association of alleles at the different loci) between some locus pairs [14], [15], [47]. As observed in this study, the stability and persistence over decades of discrete fHbp, NHBA and NadA sub/variants and non-overlapping antigen sub/variant combinations suggest they were maintained by natural selection. In the case of antigen sub/variants, the persistence was noticed also in different genetic environments such as different clonal complexes. Persistent antigen combinations in association with certain clonal complexes may indicate that acquisition of new alleles encoding antigen variants may impair fitness of that clonal complex [52].

Our observation of the persistence of the antigen sub/variants in 4CMenB may also indicate that the vaccine will be able to provide protection against populations of meningococci over time, as the antigens they target have tended to persist.

We used the Standardized Index of Association *I_A_^S^* to quantify the extent of allele association and long-term stability and f* metrics to evidence a non-overlapping structure of antigen combinations. Buckee and colleagues first used this parameter to evaluate the long-term stability of FetA and PorA combinations on carried meningococci [13]. Given the novelty of the Buckee study, our results contribute not only to an understanding of invasive meningococcal strains but also to the development of approaches for evaluating long-term stability in bacterial populations over time.

In the cc41/44 complex, the predominance of antigen sub/variants and STs shifted over time. This clonal complex is of additional public health interest because cc41/44 strains are almost equally often isolated from healthy carriers and from cases of invasive disease [48], [49], [50], [51]. Recently, a cc41/44 outbreak in the city of Aachen, Germany and 3 neighboring counties (Greater Aachen) has been described [50]. Strains of cc41/44 are also among the most important causes of serogroup B disease in the USA [51]. The central STs of cc41/44, that is to say ST-41 and ST-44, are putative ‘ancestral genotypes’ [6]. In the Netherlands strain collection, the ST-44 sub-complex was predominant during the 1960s, whereas the ST-41 sub-complex became predominant from 1980 onwards, a circumstance also observed in Belgium [48] and New Zealand [49] from 1990 onwards. As previously published, cc41/44 isolates collected during 1980 and later harbored the restriction modification system *nmeSI* [52]. In contrast, 80% of the isolates of the ST44 sub-complex appeared to have two different genes at the genomic position of the *nmeSI* system encoding a MoxR like AAA ATPase and a protein with a Von Willenbrand domain, respectively (data not shown). In eBURST analyses, fHbp variant distribution changed with the relative predominance of the ST-44 and ST-41 sub-complexes. In 1960-1970, when the ST-44 sub-complex was predominant, fHbp-2 and fHbp-3 were more common in the strain collection. After 1980 ST-41 and ST-41 associated fHbp-1 became most prevalent. Although it increased in prevalence over time, fHbp-1 was the only variant that was present in both sub-complexes at all time periods. Two additional surface proteins, PorA and FetA showed a similar trend to fHbp. NHBA-2, the most frequent sub/variant in cc41/44, was also observed in both sub-complexes at all time periods. Given the intrinsic potential variability of NHBA, the maintenance of the same sub/variant in both sub-complexes over fifty years, despite changes in the genotype and in the predominance of the other protein variants, could be a consequence of selective pressure or fitness constraints.

We examined the broadest collection of pathogenic meningococcal isolates over the longest time span available globally. Significantly, we confirmed that the sequence conservation of specific fHbp, NadA, and NHBA sub/variants observed across strains and geographic regions in recent years has also been present over the last several decades. Thus, the selection of fHbp, NadA, and NHBA as antigens in 4CMenB is supported by current and past molecular epidemiology. The hypothesis that the stability of certain sub/variants and combinations of fHbp, NadA, and NHBA likely results from natural selection also supports earlier interpretations that these proteins contribute to meningococcal survival and pathogenesis or fitness [37], [42], [53], [54], [55]. Further, our results may help to support the long-term validity of fHbp, NHBA and NadA characterization and additional typing systems for meningococci currently being implemented.

Further studies are needed in order to verify whether the observations in this strain panel are generalizable. To our knowledge, no similar panels, composed of invasive meningococcal isolates collected over a so long time period, exist in other countries. However, molecular typing data suggest that the distribution of clonal complexes in Europe shows only limited variation between individual countries [9]. Further work will also be needed to articulate the results of the present study with ongoing efforts to evaluate the clinical effects and evaluation of the strain coverage of 4CMenB [56]. The Meningococcal Antigen Typing System (MATS) has been recently described as a qualitative and quantitative assay to predict 4CMenB vaccine coverage. This assay measures for each strain the expression level and the cross-reactivity of each vaccine antigen [23]. It will be important in future to apply MATS to old and new isolates to evaluate the temporal dynamics of changes in epidemiology and of potential antigenic shift for the vaccine-target antigens in normal condition and following vaccine introduction.

A limitation of the present study is that it comprises genetic data only. The study of temporal patterns of genetic associations among vaccine protein variants and MLST clonal complexes is important, even if as an initial step. Also, protein expression was evaluated for genes that have an insertion or a frame shift mutation, only. As protein expression would yield even greater differences in bactericidal titer, the evaluation of MATS results in this panel would be very interesting.

Another limitation is that only 165 invasive isolates randomly selected were tested over a period of 50 years, and how representative are the selected isolates could appear to be actually doubtful. However, the number of isolates for each clonal complex indeed reflects the relative incidence of clonal complexes and represents a substantial spectrum of different serogroup B meningococci in the Netherlands over the last decades. In addition, carriage isolates were not included. Limiting selection to pathogenic isolates may have resulted in the over-representation of hypervirulent lineages, instead of a more even balance of all meningococcal clones [57]. Moreover, we cannot comment on the two-variant fHbp vaccine currently in clinical trials because only one of the two variants, fHbp-3.45, was identified in this study. Surprisingly, only four isolates at one time point harbored this fHbp variant, whereas fHbp-1.55, was not found in the current strain panel.

Data obtained in this study highlight the importance of monitoring over time the evolutionary pattern of surface proteins included as vaccine antigens. The stability of certain sub/variants was of course observed in a pre-vaccination era, therefore in the absence of a strong immune selection against the three antigen sub/variants. It will be interesting to monitor the long-term persistence even after the introduction of the vaccine.

Stability and longevity suggest that several fHbp, NHBA and NadA sub/variants are maintained by selection despite the fact that recombination continuously generates new sub/variants. In particular, the long-term persistence of the three antigen sub/variants included in the vaccine, fHbp-1.1, NHBA-2 and NadA-3.8, may be indicative for long term broad coverage of 4CMenB.

## Materials and Methods

### Bacterial isolates, PCR amplification and gene sequencing

One hundred sixty-five meningococcal isolates collected from clinical cases (from blood or cerebrospinal fluid) in the Netherlands were randomly selected at the Netherlands Reference Laboratory for Bacterial Meningitis (NRLBM). Approximately 30 isolates were included from each decade as follows: every 2^nd^, 5^th^, 10^th^, 20^th^, 20^th^ and 10^th^ isolate were chosen of the years 1960, 1970, 1980, 1990, 2000 and 2008-2009, respectively.

Upon receipt of bacterial isolates in the NRLBM, a monoculture of the causative isolate was frozen and stored at –80°C. All isolates were passaged fewer than 5 times. All isolates were characterized by serogroup, PorA, FetA and MLST (http://pubmlst.org/neisseria/) [6], [58], [59]. The complete characterization of isolates is reported in **Table S1**. PCR templates were prepared by boiling ∼100 *N. meningitidis* colonies from chocolate agar plates in 200 µl of distilled H_2_O for 5 min, and subsequent centrifugation. 1 µl of the supernatant was used in the PCR reaction (10 µl total volume). The amplification enzyme used was AccuPrime Taq DNA Polymerase System (Invitrogen). All genes were amplified using primers external to the coding region. The primers used for PCR and sequencing are in **Table S2**. The *fHbp* gene was amplified using primers A1 and B2. PCR conditions were: 30 cycles of denaturation at 94°C for 30 s, annealing at 58°C for 30 s, elongation at 68°C for 1min. Sequences were performed using forward primers A1, 22, and reverse primers B2, 32. The *nhba* gene was amplified using primers 1 and 6. PCR conditions were: 94°C for 30 s, 55°C for 30 s, 68°C for 1min, 30 cycles. Forward sequencing primers were 1, 22, 23, 3, 84, reverse primers were 5, 6, 7, 85, 93. *nadA* PCR primers were A1 and B. PCR conditions were: 94°C for 30 s, 56°C for 30 s, 68°C for 3min, 30 cycles. Forward sequencing primers were A1, 1, 2, 3, reverse primers were B, C, 5, 6. PCR products were purified with QIAquik PCR purification kit (QIAGEN) and sequenced using the ABI 377 automatic sequencer (Applied Biosystems).

Discrimination between ST44 and ST41 subcomplexes of clonal complex41/44 was assessed by PCRs targeting MoxR like AAA ATPase and *nmeSI*, respectively. PCR to assess *nmeSI* was performed as previously described [50], [52] using primers AVDE 0712 and AVDE 0716. Part of the gene encoding the MoxR like AAA ATPase was amplified using primers AVDE 0701F and AVDE 0710. PCR conditions were: 95°C for 1 min, 58°C for 1 min, 72°C for 2 min, 30 cycles.

### Sequence analysis and measure of the associations between different loci

DNA sequences were assembled and analysed using Sequencher version 4.10.1 sequence analysis software (Gene Codes Corporation, Ann Arbor, MI USA, http://www.genecodes.com), BioEdit (developed by Tom Hall, *Ibis Biosciences*), Molecular Evolutionary Genetics Analysis (MEGA) software version 4.0 [60] and Jalview [61].

Molecular typing of fHbp, NHBA and NadA sub/variants was based on amino acid sequence determination. fHbp sub/variants were designated as variants.oxford.peptide (http://pubmlst.org/neisseria/fHbp/). NHBA sub/variants were designated as peptides. NadA sub/variants were designated as variant.peptide.

The structure of MLST clonal complexes was analysed with PHYLOViZ 1.0 based on the goeBURST algorithm [62].

The Cramer’s V coefficient [63] was used to measure association statistics with clonal complexes. The Standardized Index of Association *I_A_^S^* [46] was used to test the stability of associations between different loci. The non-overlapping structure of antigen variant combinations was measured by f* metrics [13]. The three statistical parameters V, *I_A_^S^* and f* are based on the frequency of the alleles and vary between 0 (random distribution) and 1 (perfect association or non overlapping distribution in the case of f*).

## Supporting Information

Table S1 **Characterization of the 165 clinical isolates selected for this study.** For each isolate, year of isolation, serogroup, FetA, PorA, sequence type (ST), clonal complex, fHbp (allele, sub/variant and variant), NHBA (peptide) and NadA (gene presence, sub/variant and variant) are indicated. fHbp sub/variants were designated as reported in the database (http://pubmlst.org/neisseria/fHbp/). For NHBA and NadA, an internal nomenclature was used. FS: frame-shift mutation: INS: insertion sequence *IS*1301, causing no expression of the protein.
(XLS)

Table S2 **Primers used for PCR and sequencing.**
(XLS)
